# Investigating the Immunomodulatory Impact of Fecal Bacterial Membrane Vesicles and Their IgA Coating Patterns in Crohn’s Disease Patients

**DOI:** 10.3390/ijms252313194

**Published:** 2024-12-08

**Authors:** Nader Kameli, Heike E. F. Becker, Daisy M. Jonkers, John Penders, Paul Savelkoul, Frank Stassen

**Affiliations:** 1 Department of Medical Microbiology, College of Nursing and Health Sciences, Jazan University, Jazan 6809, Saudi Arabia; 2 Health Research Center, Jazan Univesiry, Jazan 6809, Saudi Arabia; 3 Department of Medical Microbiology, Institute of Nutrition and Translational Research in Metabolism (NUTRIM), Maastricht University Medical Center+, 6229 HX Maastricht, The Netherlands; 4 Department of Gastroenterology/Hepatology, Institute of Nutrition and Translational Research in Metabolism (NUTRIM), Maastricht University Medical Center+, 6229 HX Maastricht, The Netherlands; 5 Department of Medical Microbiology and Infection Control, VU University Medical Center, 1081 HV Amsterdam, The Netherlands

**Keywords:** gut microbiota, bacterial membrane vesicles, Crohn’s disease, inflammatory responses, IgA

## Abstract

The human intestinal tract contains trillions of bacteria that coexist in a symbiotic relationship with human cells. Imbalances in this interaction can lead to disorders such as Crohn’s disease (CD). Bacteria membrane vesicles (MVs), which are released by almost all bacteria, have been demonstrated to play a crucial role in bacteria–host interactions. In this study, we assessed the physical characterizations, immunomodulatory effects, and IgA interactions of MVs derived from fecal samples of CD patients and healthy controls (HCs). MVs were isolated from the frozen fecal samples using a combination of ultrafiltration and size-exclusion chromatography. Using nanoparticle tracking analysis, we found that the MVs of the CD patients showed a significantly lower concentration compared to those of the HCs. Cryo-transmission electron microscopy revealed the larger size of the MVs in active CD (Ac-CD) compared to the MVs of remission CD (Re-CD) and HCs. Differentiated monocyte THP-1 cells released more TNF-a when exposed to MVs from the HCs compared to the CD patients. On the other hand, the MVs from the HCs and Re-CD patients but not the Ac-CD patients induced more anti-inflammatory IL-10. Intriguingly, bead-based flow cytometry analysis showed that the MVs of the HCs and Re-CD patients were more coated with IgA compared to those of the Ac-CD patients. These results suggest the potential role of MVs in the immunomodulatory impact on the pathophysiology of CD. Moreover, IgA seems to regulate these effects by direct binding, which was not the case for the Ac-CD patients. Finally, the IgA coating patterns of the MVs could be used as an additional disease biomarker, as they can clearly identify the exacerbation status of CD.

## 1. Introduction

The intestinal tract contains trillions of bacteria that normally maintain a symbiotic situation with their host, with both parties obtaining mutual benefits [1]. Disturbances in this relationship can lead to intestinal disorders, such as Crohn’s disease (CD) [2,3]. The importance of microbiota–host interactions in maintaining health status and in the pathogenesis of diseases gives reason for investigating the mechanisms underlying the exchange of signals between the microbiota and the host. Bacteria have evolved complex pathways to deliver a variety of molecules to come in direct contact with host cells [4]. In recent years, it has been recognized that intercellular signals are largely mediated through the secretion of extracellular vesicles [5]. It is well established that, similarly to eukaryotic cells, bacteria are also able to deliver extracellular vesicles from their membranes, including a variety of signaling molecules. These spherical particles with a diameter of 50–250 nm, named membrane vesicles (MVs), contain a variety of molecules, including lipopolysaccharides (LPSs) as part of the Gram-negative bacterial outer membrane, proteins mediating adhesion and invasion to host cells, toxic and immunomodulatory compounds, cytoplasmic and inner membrane proteins, ATP, and even RNA and circular or linear DNA [6,7,8]. Studies have investigated MVs isolated from selected bacterial taxa of the human commensals regarding their function, immunoreactive potential, and inter-vesicular interactions [9,10,11]. MVs have long been recognized for their immunoreactive potential, and recent studies investigating their immunoregulatory role have determined their pro- and anti-inflammatory effects [9,10,11,12,13]. In addition, alterations in gut-derived MVs have been associated with inflammatory bowel diseases and other gastrointestinal disorders [14,15]. Interestingly, studies have indicated that differential inflammatory responses were observed when MVs from one bacterial taxon were exposed to cells in the presence of MVs from another bacterial taxon [9]. This suggests that the immunoreactive implications of bacterial MVs are dependent on their heterogenous network and that the complex underlying interactions, rather than independent players, might explain their involvement in pathologies. Despite the strong indications of the immunoregulatory involvement of MVs within the gastrointestinal microbiota, studies that investigate a heterogenous MV population from the unreserved microbiota are currently lacking.

IgA is considered a first line of immune protection at the mucosal surface. Remarkably, this protection occurs in a non-inflammatory manner, which leads to sustainable host–microbe mutualism [16,17]. IgA has also been found to play a big role in shaping the gut microbiota composition and promoting bacteria symbiosis. Commensal coating with SIgA has been correlated with the development of certain diseases, such as celiac disease, Crohn’s disease, asthma, and allergies [18,19,20]. Both increasing and decreasing levels of coated bacteria are associated with the onset of disease in all of these inflammatory disorders.

In the present study, we investigated the immunogenicity of heterogenous MV populations derived from CD patients (active and in remission) and correlated them to the IgA coating patterns of these vesicles in comparison to MVs derived from healthy controls.

## 2. Results

### 2.1. Patient Characteristics

Table 1 shows the general characteristics of both the CD patients and the healthy controls. No significant differences were seen between the groups. Most of the CD patients belonged to the B1 phenotype, with 66.7% and 83.3% in the active and remission groups, respectively (Table 1). Importantly, most patients used antibiotics or immunosuppressants, while none of the healthy controls used any of these drugs. Moreover, one participant from each group (HC, Ac-CD, and Re-CD) was on an alternative diet.

### 2.2. MV Characterizations

**Figure 1:**
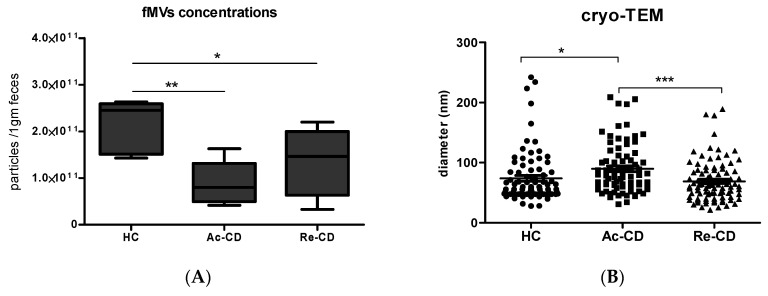

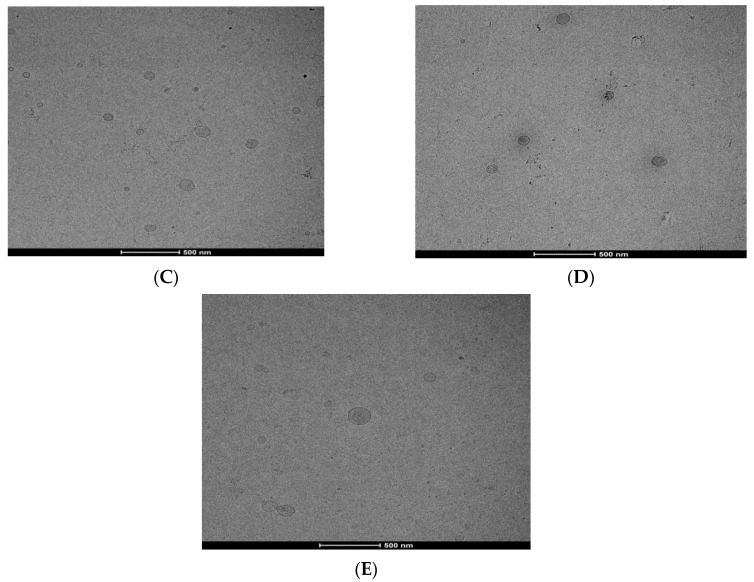
Characterization of the MVs derived from the fecal samples of the HCs and Ac-CD and Re-CD patients. (**A**) The concentrations of MVs derived from 0.5 gm of feces of all groups; data are means ± SEMs. (**B**) The size distribution of the MVs, showing significant differences in the particle sizes. The cryo_TEM images show the structure and size of the MVs in all three groups: HC (**C**), Re-CD (**D**), and Ac-CD (**E**). An unpaired *t*-test was used for statistical purposes. * *p*-value < 0.05, ** *p*-value > 0.01, and *** *p*-value > 0.001.

The MVs were characterized based on their protein concentration and particle quantification, followed by flow cytometry and transmission electron microscopy (TEM) visualization, as previously shown [21]. Quantification of the MVs was carried out using nanoparticle tracking analysis (NTA). The concentration of vesicles was higher in the healthy controls compared to the CD patients, as shown in Figure 1A. The particle concentrations from the healthy volunteers ranged from 1.43 × 10^11^ to 2.63 × 10^11^ particles/1 g of fecal sample, with a median of 2.45 × 10^11^; in the Re-CD patients, the particle concentrations ranged from 4.14 × 10^10^ to 1.63 × 10^11^ with a median of 1.47 × 10^11^; in the Ac-CD patients, the particle concentrations ranged from 3.29 × 10^10^ to 2.2 × 10^11^, with a median of 7.99 × 10^10^.

Next, the particle size distribution of the MVs from the pooled samples was determined using cryo-TEM (Figure 1C–E). Totals of 80–90 vesicles (representing two samples) were measured from each group (HC, Ac-CD and Re-CD) using ImageJ V1.53 software (Figure 1B). The mean particle size of the HC vesicles was 73.9 ± 4.7 nm (mean with SEM), with most particles being <70 nm in size. The vesicles derived from the Re-CD samples were similar in size compared to those from the HCs, with a mean of 69 ± 3.5 nm and most particles being less than 70 nm. However, in the Ac-CD samples, the mean size of the particles was 90 ± 3.5 nm, with most >70 nm (Figure 1B). The size of the particles was significantly higher in the Ac-CD group compared to the HC and Re-CD groups.

### 2.3. Inflammatory Response and Mechanism of Stimulations

**Figure 2:**
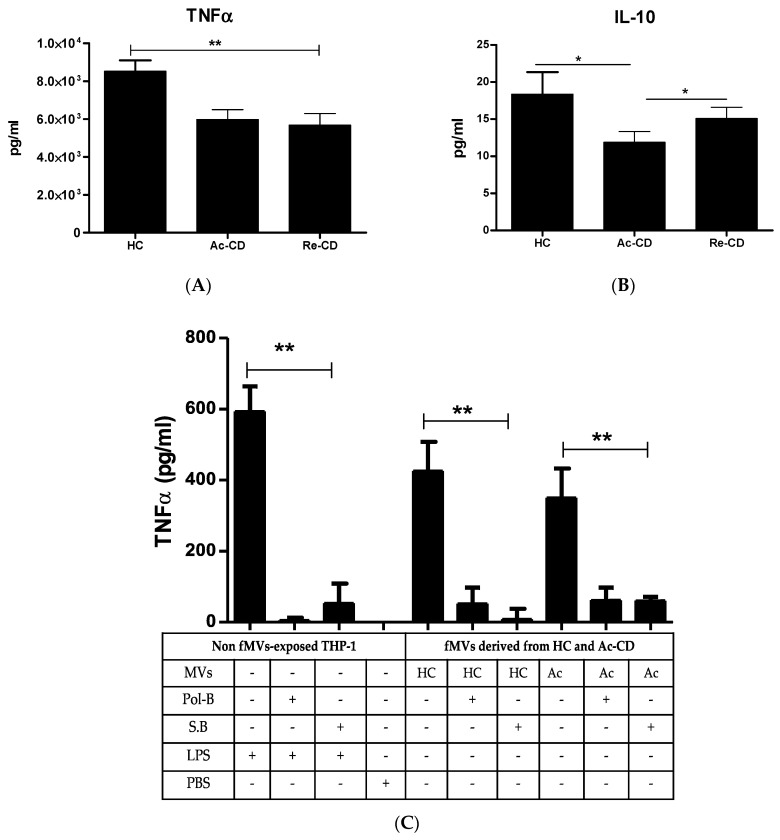

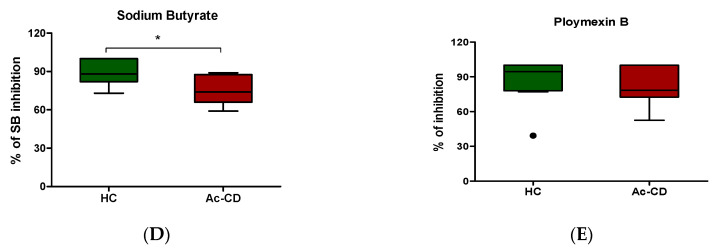
Immunoreactivity of MVs: Comparisons of cytokines released by THP-1 cells exposed to fMVs in all three groups, using TNF-α as pro-inflammatory and IL-10 as anti-inflammatory mediators (**A**,**B**), with 10^8^ particles/well. The effects of polymyxin-B (Pol-B) and sodium butyrate (SB) on the TNF-α production of THP-1 cells exposed to MVs (10^7^/well) from HCs and Ac-CD patients. LPS and PBS were used as positive and negative controls (**C**), respectively. The percentages of inhibition for both the SB (**D**) and Pol-B (**E**) inhibitors on the TNF-α released from THP-1 cells as a result of MV exposure. The bars represent the means ± SEMs. The plot graphs are displayed as whisker plots based on Tukey’s test. * *p*-value < 0.05, ** *p*-value > 0.01.

The immunoreactive effects of the MVs from the CD patients and HCs were assessed by measuring the most common inflammatory cytokines released by microphages: TNF-α as pro-inflammatory markers, and IL-10 as an anti-inflammatory marker. When exposed to a standardized concentration of 1 × 108 particles/mL for 24 h, the THP-1 cells released substantial amounts of the pro-inflammatory cytokine TNF-α and, to a lesser extent, the anti-inflammatory cytokines IL-10 (Figure 2A–C). First, we compared the inflammatory response between the CD patients and the healthy controls. Remarkably, we found significantly elevated TNF-α levels in the HC group compared to the active and remission CD groups. In contrast, IL-10, which is an anti-inflammatory cytokine, was significantly higher in the HC and remission CD groups.

In an attempt to elucidate an underlying mechanism for the induction of this pronounced TNFα response, we incubated fecal MV isolates with polymyxin B, an antibiotic that binds the lipid A region of LPS, or sodium butyrate (SB), which suppresses the LPS-induced phosphorylation of the AKT and NF-κB p65 signaling pathways in macrophages. The incubation of the MVs with polymyxin B or SB prior to exposure significantly blunted the TNFα response from THP-1 macrophages in both the active CD patients, from 350 ± 82 pg/mL to 62 ± 35.2 pg/mL and 60.8 ±10.8 pg/mL (mean ± SEM), respectively, and the HCs, from 425.9 ± 81.5 to 52.5 ± 45.3 and 8.7 ± 29.8 pg/mL (*p* < 0.01; Figure 2C). Interestingly, the percentage of inhibition was significantly reduced in the Ac-CD patients compared to the HCs (Figure 2D,E). To demonstrate the specificity of polymyxin B and SB in inhibiting LPS-induced TNFα, we also incubated LPS with polymyxin B or SB, which abolished the TNFα response to THP-1 cells from 593 ± 70 pg/mL to 5.5 and 54 pg/mL, respectively (Figure 2C).

### 2.4. MVs Coated with IgA

**Figure 3:**
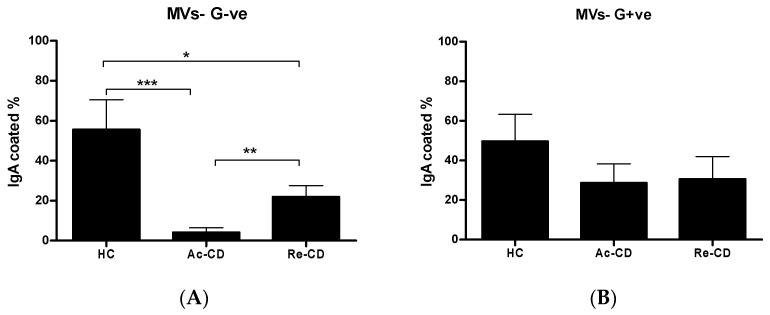
The percentages of positive IgA-coated populations among the MVs derived from the HCs, Ac-CD patents, and Re-CD patients. Selected G-ve (**A**) and G+ve (**B**). The MVs from the HCs were significantly more coated compared to those from the CD patients. Moreover, the MVs from the Re-CD patients were more highly coated with IgA than those from the Ac-CD patients. The data are presented as the means ± SEMs. * *p*-value < 0.05, ** *p*-value > 0.01, and *** *p*-value > 0.001.

To determine the initial interaction of gut microbiota-derived MVs with IgA, a coating study was conducted. As previously mentioned, MV populations were selected based on their bacterial origin types, specifically as either Gram-positive (G+ve) or Gram-negative (G-ve) bacteria. Surprisingly, the MVs derived from the HCs had significantly higher levels of IgA coating compared to those from the Ac-CD and Re-CD groups for both types of bacteria-derived MVs. The percentages of positive beads in the Gram-negative MV population were 55.52 ± 15.04% (mean ± SEM) in the HCs, 21.88 ± 5.6% in the Re-CD patients, and 4.05 ± 2.42% in the Ac-CD patients (Figure 3A). Meanwhile, the percentages in the Gram-positive population were 49.63 ± 13.67% among the HCs, 30.65 ± 11.32% in the Re-CD patients, and 28.58 ± 9.5% in the Ac-CD patients (Figure 3B).

## 3. Discussion

In the present study, we aimed to isolate bacterial vesicles from the feces of health individual and IBD patients, specifically CD patients. Moreover, we aimed to determine their immunoreactivity and IgA coating patterns. First, we successfully isolated bacterial vesicles from healthy volunteers’ and CD patients’ feces. Isolations were confirmed using different criteria according to the MISEV 2018. Cryo-TEM, together with NTA or tunable resistive sensing pulse (TRSP), FACS, and functional aspects emphasized the presence of bacteria-derived MVs [21]. Here, we provide evidence that bacterial MVs derived from the feces of healthy volunteers and CD patients are capable of evoking pronounced pro-inflammatory and, although to a lesser extent, anti-inflammatory responses in THP-1. We also showed for the first time that MVs derived from fecal samples are coated with IgA in correlation with disease progression.

In the current study, we found indications that fecal MVs isolated from CD patients may significantly differ in concentration (Figure 1). This could be due to the loss of bacterial diversity and shifts in bacterial composition, as previously observed in IBD patients [14,15,22]. As a consequence of dysbiosis, bacterial strains that contribute an immunomodulatory MV population, as described by Kang et al., could be reduced in IBD patients [9,14]. This suggests that a bacterial MV diversity may contribute to the disease progression of IBD patients through the loss of important modulators of inflammation. Furthermore, the cryo-TEM images showed various MVs sizes, which indicate different population and bacterial origins of the HC, Re-CD, and Ac-CD groups, and subsequently different immunomodulators, as previously mentioned, which we also observed in our previous studies [14,23].

Our findings concerning the role of vesicles derived from gut microbiota complement several works on the specific taxa of commensals and their role in disease initiation and progression [9,12,24]. Both anti- and pro-inflammatory effects of different commensal-derived vesicles have been demonstrated. Moreover, MVs have been found to provide protection against colitis induction by strengthening the epithelial intestinal barrier [9]. The approach we describe explains the overall impact of fecal-derived vesicles on inflammatory responses. TNF-a released by exposed THP-1 cells was expected to be at a higher level in the CD patients’ samples; however, in this study, it showed lower values compared to the HC samples. This might be due to the high concentration of bacterial-derived vesicles in the HCs compared to the Ac-CD and Re-CD patients. On the other hand, anti-inflammatory marker IL-10 was significantly higher in the HC and Re-CD samples, which indicates the higher immunomodulatory impact of these populations (Figure 2). In our experiments, polymyxin B, an antibiotic that binds to the lipid A region of LPS, significantly blunted the MV-mediated TNF-α response induced by THP-1 cells, suggesting a TLR4-mediated induction of TNF-α as a main part of the underlying mechanism [25]. Yet, the inability of polymyxin B to completely abolish the TNF-α response induced by fecal MV strongly indicates that mechanisms other than LPS/TLR4, to a lesser extent, also mediate these immune responses in THP-1 macrophages (Figure 2). Moreover, the reduction in released TNF-a was not significantly different between the HC and Ac-CD groups. In order to detect the pathway of TNFa production, sodium butyrate (SB) was used, which inhibits the LPS-induced phosphorylation of the NF-κB p65 and AKT signaling pathways [26]. The SB-treated cells showed a significant difference in the TNF-a production pathways between the HC and Ac-CD groups, which indicates that another signaling pathway, such as the MAPK pathway, could be used by Ac-CD MVs. In the future, determining the LPS content of MV samples by means of the LAL test may enable further insights into the dependency on LPS to induce a pro-inflammatory response in the cells of the innate immune system.

The coating of commensal bacteria with secretory immunoglobulin A (SIgA) has been implicated in the pathogenesis of several diseases, including celiac disease, Crohn’s disease (CD), asthma, and allergies [18,19]. In some inflammatory disorders, lower levels of SIgA-coated bacteria have been correlated with disease onset, suggesting a potential protective role for an adequate SIgA coating. Conversely, elevated percentages of bacteria coated with IgA and IgG have been observed in patients with inflammatory bowel disease (IBD), particularly those with Crohn’s disease [27].

Furthermore, studies in murine models have provided direct evidence linking SIgA-coated bacteria to disease progression. For instance, IgA-coated bacteria derived from colitis patients have been shown to include colitogenic intestinal bacteria capable of inducing intestinal inflammation [28]. When germ-free mice were colonized with highly IgA-coated bacteria, their susceptibility to IBD significantly increased as compared to mice colonized with bacteria that exhibited lower levels of IgA coating [28]. We have previously developed a bead-based flow cytometry assay to specifically select for either Gram-negative or -positive-derived MVs [21]. This approach allows for determining the IgA coating patterns of select MV populations. Although studies have indicated that IgA-coated bacteria are increased in IBD disease [27], the presence of LPS enables MVs derived from Gram-negative bacteria to engage with immune cells and modulate inflammation. Studies have demonstrated that IgA can coat commensal bacteria, targeting them for recognition using mucosal immune cells, facilitating their clearance, and supporting immune regulation at mucosal surfaces [29]. This coating allows the host immune system to efficiently neutralize bacterial antigens carried within the vesicles, preventing the dissemination of pathogenic factors. In the current study, the IgA coating pattern was more predominant in the HCs for both gram-negative and gram-positive derived vesicles and, to a lesser extent, in the Re-CD patients. These results are supported by the IgA deficiency that is frequently found in IBD patients and the IgA-degrading capabilities of *Sutterella* species, which are more prevalent in IBD patients compared to healthy individuals [6,30,31]. In alignment with previous studies, there are two suggested mechanisms of IgA coating. First, IgA may protect the crossing of MVs through the intestinal barrier; second, it may regulate the role of these vesicles in host–bacteria interactions or bacteria–bacteria interactions [32,33]. Altogether, the impact of IgA coating patterns may explain the high production of TNF-α in the HCs compared to the CD patients, as MVs in healthy people do not have access and cannot reach immune cells in the lamina propria. In conclusion, membrane vesicles from both Gram-positive and Gram-negative bacteria are critical players in bacterial–host interactions, with IgA coating representing a key mechanism of immune regulation at mucosal surfaces. Ongoing research into these interactions is essential for understanding bacterial pathogenesis and developing novel therapeutic interventions aimed at enhancing mucosal immunity.

The limitation of the present study is the relatively small sample size, as only 12 CD patients were tested. It would also be of great interest to sort the highly IgA-coated vesicles and determine their bacterial origins and their immunomodulatory effects. Broad pro- or anti-inflammatory cytokine detection is highly recommended to obtain an overview of different immunoreactivity. Proteomics analysis will provide in-depth information on these vesicles that may help further to understand the particular structures involved in their immunomodulation. Nevertheless, our study provides a foundation for further understanding how gut microbiota-derived vesicles, as a mixed population, interact with the immune system.

In conclusion, the present study highlights the potential immune-modulatory role of fecal-derived membrane vesicles and their IgA coating in IBD pathogenesis. In addition, IgA coating patterns can be used as a non-invasive method for the diagnosis of active and remissive states of Crohn’s disease.

## 4. Materials and Methods

### 4.1. Study Population

Fecal samples were collected from 12 patients with Crohn’s disease (either with active disease, *n* = 6, or in remission, *n* = 6) in the IBD South Limburg (IBD-SL) biobank project [34]. Samples from 6 healthy volunteers in the Maastricht IBS Cohort (MIBS) were also included in this study. Crohn’s disease was diagnosed based on clinical and endoscopic or radiological findings conforming to the ECCO guidelines [35]. The fecal samples were collected by the patients at home, stored at 4 °C, and brought to the hospital within 2 h after defecation. The samples were aliquoted and frozen directly at −80 °C for further analysis. Disease activity was defined using the simple endoscopic score (SES-CD) [36]. Active disease was defined at an SES-CD score of ≥3, while remission was defined at an SES-CD score of <3.

### 4.2. Vesicle Isolation from Fecal Samples

The steps for optimizing a protocol for isolating heterogeneous MV populations from the fecal samples (fMVs) were based on previous research performed by Benedikter et al. [37], in which a protocol was designed to isolate MVs from cell culture media, which was modified by our group for feces MVs [21]. Briefly, approximately 0.5 g of fecal matter was dissolved in 10 mL of filtered phosphate buffered saline (PBS). Two rounds of centrifugation were carried out (15 min, 5000 rpm, 4 °C) to remove solid debris and cells. Next, an additional ultracentrifugation step (2.5 h, 40,000 rpm, 4 °C) was carried out on the supernatant, followed by filtration over 0.45 μm (Acrodisc syringe filters, Pall Life Sciences, AZ, USA) and 0.2 μm (Minisart© NML syringe filter, Sartorius Stedim Biotech, Göttingen, Germany) filters. To separate the vesicles from the molecules, the filtrate was then loaded onto a filter with a molecular weight cut-off of 100 kDa (Amicon Ultra 15 mL Centrifugal Filter Unit, Merck Millipore, Billerica, MA, USA) and concentrated to 250 μL using centrifugation (45 min, 4000 rpm, 4 °C). The filter membrane was additionally rinsed with 250 μL of sterile PBS in order to achieve complete MV recovery, and a final volume of 500 μL was used for the next step.

The next step involved the purification of the concentrate by separating the vesicles from the free protein. This was achieved using size-exclusion chromatography (SEC) with 10 mL sepharose columns (GE Healthcare, Eindhoven, the Netherlands). The concentrated supernatant was loaded onto the column, and fractions of 0.5 mL were collected in Eppendorf tubes. In total, 24 fractions of 0.5 mL were collected per sample. Fractions 7–11, which contained membrane vesicles, were pooled and quantified using a nanoparticle tracking assay (NTA) (ZetaView). The pooled fractions were stored at −80 °C until further analysis.

### 4.3. Visualizing MVs Using Electron Microscopy (Cryo-TEM)

Three microliters of isolated vesicles were applied to a glow-discharged holey carbon grid before blotting against filter paper to leave only a thin film spanning the grid holes. The sample was kept at 95% humidity before plunge-freezing in liquid ethane using a Vitrobot (FEI, Eindhoven, The Netherlands). The vitreous sample films were transferred to a Tecnai Arctica Cryo-Transmission Electron Microscope (ThermoFisher, Bleiswijk, The Netherlands). Images were taken at 200 kV with a Falcon camera (ThermoFisher, The Netherlands).

### 4.4. Immunogenicity of fMVs

#### 4.4.1. Cell Culture

The human monocytic THP-1 cell line (ATCC-TIB202) was maintained in RPMI1640 (Sigma, St. Louis, MO, USA) supplemented with 10% FCS (Lonza, Verviers, Belgium), glucose (22.5%), sodium pyruvate (100 mM), and β-mercaptoethanol (25 mM) and cultured at 5% CO_2_ and 37°C. For monocyte differentiation, the cells were seeded at 1 × 10^4^ cells/wells in a 96-well plate and stimulated for 48 h with 100 nM phorbol 12-myristate 13-acetate (PMA; Sigma, St. Louis, MO, USA).

#### 4.4.2. Stimulation of THP-1 Cells

After differentiation, all PMA-containing medium was replaced for 24 h with normal culture medium, and the cells were stimulated for 24 h, with 10^8^ particles/mL of feces-derived vesicles. As a negative control, other cells were left unstimulated. LPS (1 mg/mL) was used as a positive control for the experiments. After 24 h, the supernatants were collected and centrifuged for 10 min at 1200 rpm to remove cells/debris and subsequently stored at −20 C for future cytokine analysis. The final concentrations in the well were as follows: polymyxin B—25 μg/mL; SB—2.5 mM; LPS—10 μg/mL.

#### 4.4.3. Release of Cytokines by THP-1 Following fMV Exposure

As indicators for the immunoreactive potential of the MVs, we measured the release of pro-inflammatory (TNF-α) and anti-inflammatory (IL-10) markers (enzyme-linked immunosorbent assay; Human Ready-Set-Go ELISA kit, Affymetrix eBioscience; 88-7346-86, 88-7066-77, 88-7106-77) using the manufacturer’s instructions. All samples, including the positive and negative controls, were diluted to within the measurement ranges of 1:20, and 1:4 for TNF-α and IL-10, respectively. The optical density was measured at 450 nm–570 nm, and the delta OD was obtained using a Biotek Instruments Powerwave X Microplate Reader.

### 4.5. Flow Cytometry Analysis for IgA Coating

#### 4.5.1. Preparation of Antibody-Coated Latex Aldehyde Beads

Previously, we described this method to determine the presence of MVs released by different bacterial species (e.g., *P. aeroginosa* and *M. catarrhalis*) [38]. In this study, we slightly adapted this method to determine the presence of MVs in fecal samples [21]. Briefly, a total of 1 × 10^8^ 4 µM aldehyde–sulfate beads were washed in 150 µL MES buffer (all washing steps were performed at 3000× *g* for 10 min) and coated with 25 µg antibody, either against outer membrane protein A (ompA) for Gram-negative bacteria, or against lipoteichoic acid (LTA) for Gram-positive bacteria, overnight at 4 °C, while keeping a total 170 µL of solution under constant agitation at 6500 rpm. After coating, the remaining free binding sites on the beads were blocked by washing the beads 3 times with 0.22 µM filtered PBS with 4% (*w*/*v*) bovine serum albumin (BSA). Then, the beads were resuspended and kept in a total of 500 µL storage buffer (PBS with 0.1% (*v*/*v*) glycine and 0.1% (*w*/*v*) sodium azide) at 4 °C.

#### 4.5.2. Bead-Based Flow Cytometry for the Detection of IgA Coatings of fMVs

MVs were incubated with the coated beads overnight at room temperature under constant agitation at 6500 rpm. After overnight vesicle capture, the beads were washed twice with 0.22 µM filtered PBS with 2% (*w*/*v*) BSA, followed by a final incubation with IgA secondary PE-conjugated antibody (Miltenyi Biotec, Bergisch Gladbach, Germany) for 1 h at room temperature under constant agitation with light protection. Then, the beads were washed twice in 0.22 µm filtered PBS with 2% (*w*/*v*) BSA, after which, the pellets were re-suspended in 150 µL of PBS and analyzed on a FACS Canto^TM^ (BD Bioscience, Franklin Lakes, NJ, USA). Analyses were performed using FACS Diva V8.0.1 Software. Single beads were gated according to their forward and side scatter. The quartile distribution within a dot plot, based on the fluorescent intensity of single beads, was then used to calculate the relative fluorescent intensity unit. The quartile of gate 4 was set to 2% for beads incubated with PBS. The IgA-coated MV population was determined based on the percentage of positive beads in the quartile of gate 4.

### 4.6. Statistical Analyses

GraphPad Prism 5 Software (GraphPad, San Diego, CA, USA) was used for all analyses. An unpaired *t*-test was performed for the statistical analysis of variance between the means of 2 groups. Differences were considered significant when *p* ≤ 0.05.

## Figures and Tables

**Table 1:** Baseline characterizations of the samples: healthy controls (HCs) and Crohn’s disease patients (CDs).

	CD [12]		HC (6 Samples)
	Active (6 Samples)	Remission (6 Samples)	
Gender (male)	3	1	1
Age: Median (range)	47.5 (25–63)	49.5 (38–67)	41.5 (25–50)
Smoking:			
Never (N)	3 (N)	3 (N)	3 (N)
Yes (Y)	1 (Y)	2 (Y)	1 (Y)
Stop > 6 month (>6)	1 (>6)	1 (>6)	2 (>6)
Stop < 6 month (<6)	1 (<6)		
Age at diagnosis:			NA
A1: <16 y	1 (A1)		
A2: 17–40 y	3 (A2)	4 (A2)	
A3: >41 y	2 (A3)	2 (A3)	
Disease phenotype:			NA
B1: Non-structuring/nonpenetrating	4 (B1)	5 (B1)	
B2: Structuring	1 (B2, B3)		
B3: Penetrating	1 (B3)	1 (B3)	
Disease location:			NA
L1: Ileal	3 (L1)	2 (L1)	
L3: Ileocolonic	1 (L2)	1 (L2)	
L2: Colonic	2 (L3)	3 (L3)	
Medication use:			No
Antibiotics	2	1	
Immunosuppressant	5 (Biological)	4 (Azathioprine, methotrexate)	
SES-CD score:		6 remissions	NA
0–2: Remission			
3–6: Mild endoscopic activity	2 (Mild)		
7–15: Moderate endoscopic activity	3 (Moderate)		
>15: Severe endoscopic activity	1 (Severe)		

## Data Availability

This study’s original contributions are included in the article. For further inquiries, please contact the corresponding authors.
